# Mid-Luteal 17-OH Progesterone Levels in 614 Women Undergoing IVF-Treatment and Fresh Embryo Transfer—Daytime Variation and Impact on Live Birth Rates

**DOI:** 10.3389/fendo.2018.00690

**Published:** 2018-11-29

**Authors:** Lise Haaber Thomsen, Peter Humaidan, Karin Erb, Martin Overgaard, Claus Yding Andersen, Ulrik Schiøler Kesmodel

**Affiliations:** ^1^The Fertility Clinic, Skive Regional Hospital, Skive, Denmark; ^2^Department of Clinical Medicine, Aarhus University, Aarhus, Denmark; ^3^The Fertility Clinic, Odense University Hospital, Odense, Denmark; ^4^OPEN, Odense Patient Data Explorative Network, Odense University Hospital, Odense, Denmark; ^5^Department of Biochemistry, Odense University Hospital, Odense, Denmark; ^6^Laboratory of Reproductive Biology, The Juliane Marie Centre for Women, Children and Reproduction, University Hospital of Copenhagen, University of Copenhagen, Copenhagen, Denmark; ^7^The Fertility Clinic, Herlev University Hospital, Herlev, Denmark; ^8^Department of Clinical Medicine, University of Copenhagen, Copenhagen, Denmark

**Keywords:** 17-OH progesterone, progesterone, IVF, live birth, daytime variation

## Abstract

**Introduction:** Corpus luteum (CL) produces progesterone (P_4_) and 17-OH progesterone (17-OH P_4_) during the luteal phase. Contrary to P_4_, 17-OH P_4_ is not supplied as part of the luteal phase support following IVF-treatment. Therefore, measuring endogenous serum 17-OH P_4_ levels may more accurately reflect the CL function compared to monitoring serum P_4_ concentrations.

**Objective:** To explore the correlation between mid-luteal serum 17-OH P_4_ levels and live birth rates and to explore the possible daytime variations in mid-luteal serum 17-OH P_4._

**Design:** Prospective cohort study.

**Patients:** 614 women undergoing IVF-treatment and fresh embryo transfer.

**Intervention:** All patients had serum 17-OH P_4_ measured 7 days after oocyte pick-up (OPU+7). Furthermore, on OPU+7, seven patients underwent repeated blood sampling during daytime to clarify the endogenous daytime secretory pattern of 17-OH P_4_.

**Outcome measure:** Live birth rate.

**Secondary outcome measure:** Daytime variation in serum 17-OH P_4_ levels.

**Results:** The highest chance of a live birth was seen with mid-luteal 17-OH P_4_ between 6.0 and 14.0 nmol/l. The chance of a live birth was reduced below (RD −10%, *p* = 0.07), but also above the optimal range for 17-OH P_4_ (RD −12%, *p* = 0.04). Patients with diminished CL-function (17-OH P_4_ < 6 nmol/l) displayed clinically stable 17-OH P_4_ values, whereas patients with 17-OH P_4_ levels >6 nmol/l showed random 17-OH P_4_ fluctuations during daytime.

**Conclusion:** The association between 17-OH P_4_ and reproductive outcomes is non-linear, and the negative effect of excessive CL-secretion seems to be just as strong as the negative effect of a reduced CL-function during the peri-implantation period.

## Introduction

Following ovulation, the human corpus luteum (CL) produces progesterone (P_4_) and 17-OH progesterone (17-OH P_4_) upon stimulation with luteinizing hormone (LH) or human chorionic gonadotropin (hCG). Progesterone governs the secretory transformation of the endometrium prior to implantation and an adequate luteal P_4_ level is crucial for the establishment and maintenance of early pregnancy (1).

During IVF and fresh embryo transfer, the luteal function is disrupted and the success of the treatment is critically dependent on exogenous luteal phase support (2–5). For decades, this exogenous P_4_ support has been administered as a standard dose in IVF patients in the firm belief that “one dose fits all.” A widely held view has been that the absolute luteal P_4_ level does not affect the chance of pregnancy, as long as a minimum P_4_ concentration was reached by means of the administration of exogenous luteal phase support (6, 7). However, recent studies have suggested that both very low and very high luteal P_4_ levels affect the reproductive outcome negatively (8–12). Thus, in a study by Yovich et al., 529 artificial frozen-thawed cycles with single blastocyst transfer were evaluated (8). The authors reported that the optimal pregnancy and live birth rate was achieved when mid-luteal serum P_4_ was in the range of 70–99 nmol/l. Below, but also above this range, the clinical pregnancy rate was significantly reduced from 64 to 44%. Following this, several other papers also reported a lower, as well as, higher luteal P_4_ threshold in artificial frozen-thawed embryo transfer cycles (10–12). In IVF cycles with fresh embryo transfer, the mid-luteal P_4_ requirement is significantly increased compared to both the natural and the frozen embryo transfer cycle as demonstrated by a work by Humaidan et al. (2). Very recently, our group described the optimal P_4_ levels during the early and mid-luteal phase of IVF cycles with fresh embryo transfer (9). In a cohort of 602 patients, we observed that reproductive outcomes seemed consistently decreased below, but most distinctly above a defined optimal P4 range.

Taken together, it seems that both too high and too low luteal P_4_ concentrations result in reduced pregnancy rates in both fresh and frozen embryo transfer cycles. The findings of a higher and lower P_4_ threshold seem plausible from a biological point of view: A very high P_4_ level during the early luteal phase may advance the endometrium leading to asynchrony between embryo development and endometrial receptivity, whereas a very low P_4_ level fails to support a sufficient secretory transformation in time for implantation. Both scenarios hamper the chance of a live birth.

The CL produces not only P_4_, but also 17-OH P_4_ during its lifespan (13). When measuring serum P_4_ following fresh embryo transfer with the use of exogenous P_4_ luteal support, the serum P_4_ value is a combination of the exogenously supplied P_4_ and the endogenous luteal P_4_ production. As 17-OH P_4_ is not supplied as part of the luteal phase support, the serum 17-OH P_4_ level may reflect more accurately the true CL function compared to the measurement of total P_4_.

The aim of this study was to explore the possible correlation between mid-luteal serum 17-OH P_4_ levels and the reproductive outcome in terms of live birth rates following IVF treatment and fresh embryo transfer. Furthermore, if serum 17-OH P_4_ should serve as an index for CL function, it is evident that the accuracy of a single measurement is important. Therefore, a second aim of the present study was to explore the daytime variations in serum 17-OH P_4_ which might affect the clinical interpretation of the measurement.

## Materials and methods

### Study design

Prospective cohort study.

### Patient population

The present cohort of patients has previously been described in papers by our group (9, 14). Briefly, this study included 614 patients undergoing IVF treatment at four public Danish fertility centers—The Fertility Clinic Skive Region Hospital, The Fertility Clinic Horsens Region Hospital, The Fertility Clinic Herlev Hospital and The Fertility Clinic Odense University Hospital—between May 2014 and June 2017. The patient cohort was unselected, representing normal everyday patients treated in the clinics. All participating patients were under the age of 41 and with a body mass index (BMI) < 35 kg/m^2^ as required by Danish national guidelines for public fertility treatment^1^ Treatment choices regarding type of protocol (GnRH agonist or GnRH antagonist) and trigger type (hCG or GnRH agonist) were made on an individual basis by the attending clinician.

**Figure 1:**
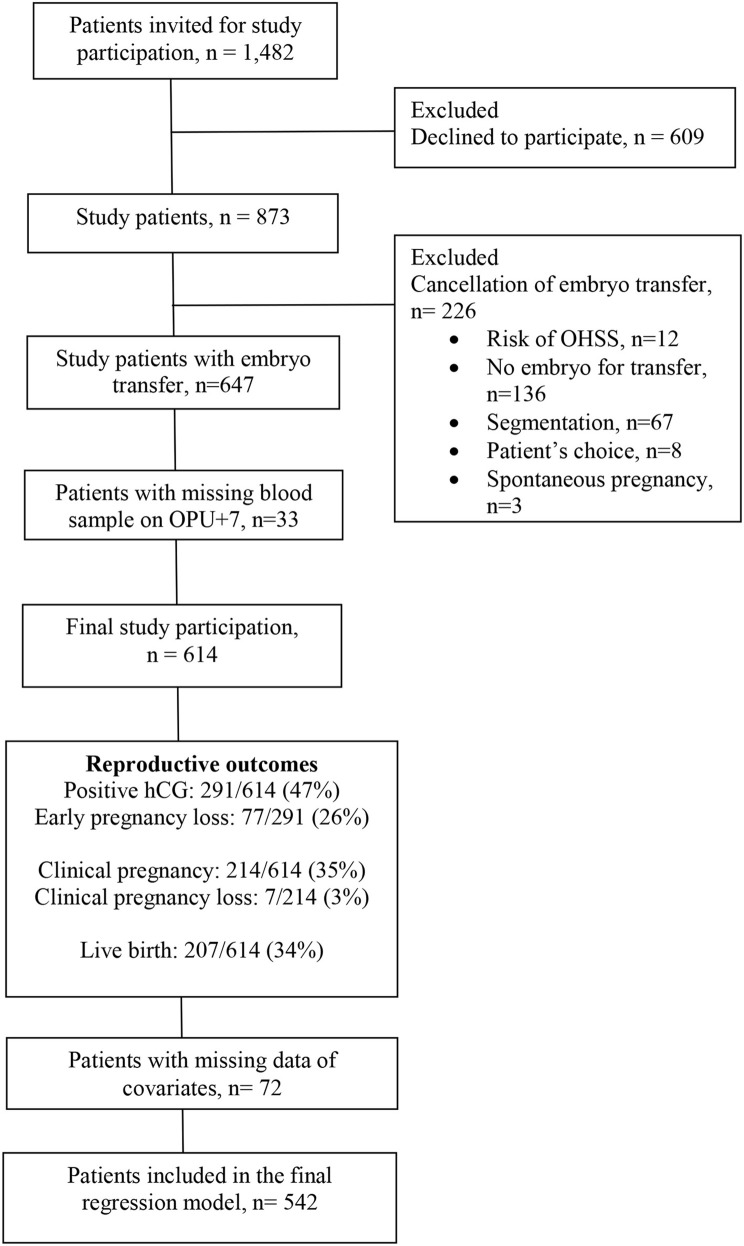
Flowchart of study participation.

Written and oral information was given to 1,482 patients of whom 609 (41%) declined to participate mainly due to the extra visit needed at the clinic for mid-luteal blood sampling 7 days after oocyte retrieval (OPU+7). The final study cohort included 614 patients with embryo transfer and relevant study samples taken (Figure 1).

Clinical information regarding primary diagnosis, age, BMI, smoking habits, antral follicle count and basal FSH and LH levels were obtained prior to treatment by the clinical staff. Serum TSH and prolactin levels were within normal range in all patients prior to treatment start. All patients participated once, only. No patients were lost to follow-up.

### Ovarian stimulation

Patients treated in the long GnRH-agonist protocol were down-regulated using daily SC injections of a GnRH antagonist (Suprefact®, Sanofi, Denmark or Gonapeptyl®, Ferring Pharmaceuticals, Denmark) starting in the mid-luteal phase of the preceding cycle and continuing until the day before ovulation induction. Ovarian stimulation was initiated after 12–14 days of down-regulation in case of an endometrial thickness <4 mm. Final follicle maturation was induced with hCG 5,000–10,000 IU (Pregnyl®, MSD, Denmark or Ovitrelle, Merck Biopharma, Denmark) when two or more leading follicles reached a diameter of ≥17 mm.

If the GnRH antagonist protocol was used, ovarian stimulation commenced on day 2 or 3 of the cycle after a vaginal ultrasound examination. Daily GnRH antagonist co-treatment was started from cycle day 6 and continued up until the day of ovulation induction. When at least two follicles reached a size of ≥17 mm, final oocyte maturation was induced with SC Buserelin 0.5 mg (Suprefact®, Sanofi, Denmark) or hCG 5,000–10,000 IU (Pregnyl®, MSD, Denmark or Ovitrelle, Merck Biopharma, Denmark).

Ovarian stimulation was performed with either hMG (Menopur®, Ferring Pharmaceuticals, Denmark), r-FSH (Gonal-f®, Merck Biopharma, Denmark), or rFSH/LH (Pergoveris, Merck Biopharma, Denmark) alone or in combination with corifollitropin-alfa (Elonva, MSD, Denmark). The initial gonadotropin dosage was determined individually based on previous response to ovarian stimulation, as well as, patient age, body mass index, antral follicle count, and basal levels of follicle stimulating hormone (FSH). Dose adjustments were performed according to ovarian response monitored by transvaginal ultrasound during treatment. Oocyte pick-up (OPU) was carried out 36 h after trigger administration. *In vitro* fertilization (IVF) or intracytoplasmic sperm injection (ICSI) was performed according to normal clinical practice. A maximum of two embryos were transferred on either days 2, 3, or 5 following oocyte retrieval.

Trained embryologists on site evaluated the quality of all available embryos. All original embryo scores from the four clinics were subsequently evaluated by two independent leading embryologists and allocated a final score from 1 to 3 (1 being a top-quality embryo, 2 being an intermediate embryo, 3 being a low-quality embryo). In case of incongruence, a second evaluation was performed to reach final agreement.

Briefly, a top-quality embryo on day 2 and 3 was described as having four and eight cells, respectively, equally sized blastomeres, <10% fragmentation and no multinucleate cells in accordance with the consensus scoring system for cleavage-stage embryos described by the Alpha Scientist group (15). In case of severe fragmentation (>25%), cell-size not stage-specific or evidence of multinucleation the cleavage embryo was classified as low-quality. The remaining cleavage embryos were classified as intermediate.

A top-quality blastocyst had a day 5 score better than 3BB according to the Gardner standard based on grade of expansion, trophectoderm, and inner-cell mass quality (16). A low-quality blastocyst had a day 5 score < 3BB. The remaining blastocysts (3BB, 4BB, 5BB) were described as intermediate.

### Luteal phase support

All patients received the same vaginal luteal phase support in a standard regimen using 300 mg micronized P_4_ daily (Lutinus®, Ferring Pharmaceuticals). Intramuscular P_4_ for luteal support was not used in any of the participating patients. A small fraction of patients (*n* = 41) had one bolus of GnRH agonist (Gonapeptyl 0.1 mg) on OPU+7 based on an individual clinical assessment. In patients receiving Gonapeptyl®, 30/41 were treated in the long GnRH agonist protocol and 11/41 in the GnRH antagonist protocol. Patients receiving a bolus of GnRH as luteal phase support were distributed equally across the different 17-OH P4 groups (*p* = 0.35).

In case of a GnRH-agonist trigger, a bolus of hCG on the day of oocyte retrieval (1,500 IU) was given to all patients. Based on the individual ovarian response to stimulation, some patients received an additional bolus of HCG on OPU+5 according to a protocol previously described by Humaidan et al. (17). Vaginal P_4_ administration continued until the day of pregnancy testing (hCG trigger) or until 7 completed weeks of gestation (GnRHa trigger).

### Blood sampling

All 614 patients had blood samples performed 7 days after oocyte pick-up (OPU+7) for hormone measurements and 14 days after oocyte pick-up (OPU+14) for pregnancy testing.

On OPU+7, seven patients agreed to have a series of blood samples performed during daytime to assess the possible variation in serum 17-OH P_4_ levels over time. These seven women were admitted to the fertility unit at Skive Region Hospital early in the morning and stayed at the clinic for the subsequent 12 h. The starting time for blood sampling was between 6 and 8 a.m. for all patients. Participants were allowed normal daily life activities during the study period. An intravenous cannula was inserted into a vein in the antecubital fossa and blood samples (4 ml) were drawn every 60 min for 12 h (*n* = 7) and for two of these hours every 15 min (*n* = 6 because of difficult venous access in one patient).

After coagulation at room temperature, all blood samples were centrifuged, and serum was isolated and divided into three separate aliquots to allow for analyses at different laboratories. Individual serum samples were stored at −80°C until analysis. Blood samples from the total cohort (*n* = 614) were analyzed for 17-OH P_4_ and P_4_, whereas the series of blood samples in the small cohort (*n* = 7) were analyzed for 17-OH P_4_ and LH.

### Hormone assays

Serum 17-OH progesterone concentrations were measured using liquid chromatography-tandem mass spectrometry (LC-MS/MS) at the Department of Biochemistry, Aarhus University Hospital, Denmark. The assay allowed quantification of 17-OH P_4_ in the range 0.37–78.7 nmol/l without dilution of samples. The accuracy was ± 0.32 nmol/l at 17-OH P_4_ concentrations of 1.3, ± 0.90 nmol/l at 17-OH P_4_ concentrations of 6.4 and ± 6.6 nmol/l at 17-OH P_4_ concentrations of 47.0 nmol/l.

Serum P_4_ and serum β-hCG concentrations were measured at the Department of Biochemistry, Odense University Hospital, Denmark using commercial automated electro chemiluminescent immunoassays (Immulite® 2000XPi, Siemens Healthcare, Denmark and Architect® i2000SR, Abbott Diagnostics, USA) routinely used for analysis. Serum LH concentrations were measured at the Department of Biochemistry, Viborg Region Hospital, Denmark, using commercial automated electro chemiluminescent immunoassays (Cobas® Modular analytics E170, Roche Diagnostics, Switzerland). The detection limit for P_4_ was 0.6 nmol/l, and the in-house inter- and intra-assay coefficients of variation were 4.4 and 1.6%, respectively. The detection limit for hCG was 1.2 IU/l and the in-house inter- and intra-assay coefficients of variation were 3.4 and 1.7%, respectively. The detection limit for LH was 0.1 IU/l and the in-house inter- and intra-assay coefficients of variation were 3.8 and 1.8%, respectively.

### Exposure

Patients were divided into four 17-OH P_4_ groups based on raw data of pregnancy outcomes: 17-OH P_4_ < 6, 6–14, 14.1–30, and >30 nmol/l (Supplementary Figure 1). The lower threshold of 6 nmol/l corresponds to the mid-luteal 17-OH P_4_ level of the natural cycle (18).

In sensitivity analyses, estimates were also calculated based on 25/50/75 percentiles, as well as, 10/50/90 percentiles (Supplementary Figure 1).

### Outcome variables

Serum β-hCG concentration was determined on OPU+14 and was considered positive if β-hCG >10 IU/l. In case of a β-hCG level between 10 and 45 IU/l, a control β-hCG was performed after 48 h. Clinical pregnancy was defined as the presence of a live fetus within an intra-uterine gestational sac at ultrasound examination in gestational weeks 7–8. Early pregnancy loss was defined as (1) patients with an insufficient β-hCG value at the day of pregnancy testing (10–45 IU/l) and decreasing β-hCG values toward null in subsequent hCG-controls (2) patients with a positive hCG but no intra- or extrauterine sac visualized on transvaginal ultrasound in gestational weeks 7–8, and (3) patients with a fetus without visible heartbeat at UL in gestational weeks 7–8. Clinical pregnancy loss was defined as the loss of a viable intrauterine pregnancy up to and including gestational weeks 20+0. Live birth was defined as the delivery of a live infant after gestational weeks 20+0. For description of gestational age, clinical gestational dating was applied using the day of oocyte retrieval as gestational weeks 2+0.

### Confounding factors

The confounding factors included in the regression model were chosen a priori based on a Directed Acyclic Graph (DAG) (Supplementary Figure 2). DAGs are visual representations of causal paths between exposure and outcome (19, 20). Drawing and analysis of a DAG can help to identify confounding factors that obscure the real effect of the exposure on the outcome. Based on a structured analysis of the DAG, it is possible to identify a minimum, however sufficient set of covariates to adjust for in the statistical analysis, which will cover all confounding elements. The web application DAGitty was used to draw and analyze the DAGs used in this paper.

### Statistical methods

Data are presented as mean and standard deviation for continuous parametric variables, percentages for categorical variables and median and range for continuous, non-parametric variables. Differences in categorical variables between 17-OH P_4_ groups were assessed with Fishers exact test or Pearson's chi-square test when appropriate. Differences in continuous parametric data between the four 17-OH P_4_-groups were assessed using one-way analysis of variance (ANOVA) followed by a *post-hoc* pairwise comparison in case of a statistical difference between groups. Normality was checked by QQ-plots, and the assumption of variance homogeneity was tested by Bartlett's test. Kruskal-Wallis test was used in case of non-parametric continuous data.

A multiple logistic regression model was used to assess the association between mid-luteal 17-OH P_4_ levels and the hCG test result (positive/negative), clinical pregnancy (yes/no) and early pregnancy loss (yes/no), and live birth (yes/no). The model included the independent variables maternal age (continuous, ln-transformed), maternal BMI (continuous, ln-transformed), smoking (yes/no), final follicle count on the day of trigger (continuous, ln-transformed), late follicular phase P_4_ level [dichotomous (>4.77 or ≤ 4.77 nmol/l)] and day of transfer [dichotomous (cleavage-stage or blastocyst)] for estimates of positive hCG rate, clinical pregnancy rate, and live birth rate. For estimates of early pregnancy loss adjustment was made for maternal age (continuous, ln-transformed), maternal BMI (continuous, ln-transformed), smoking (dichotomous), final follicle count (continuous, ln-transformed), day of transfer [dichotomous (cleavage-stage or blastocyst)] and peak estradiol level on the day of trigger (continuous, ln-transformed). The cut-off for late follicular phase progesterone (>4.77 ng/ml equivalent to >1.5 ng/ml) was chosen based on the results of earlier studies (21, 22).

In case of missing data of covariates, patients were omitted from the final regression analysis (*n* = 72). A *p* < 0.05 was considered statistically significant. All statistical analyses were performed using STATA version 13.

### Ethics

The study was conducted according to the declaration of Helsinki for Medical Research and approved by the local Ethics Committee of Central Denmark Region (M-2012-423-12). All patients gave their written and oral consent prior to study participation. ClinicalTrial.gov registration number NCT02129998.

## Results

### Demographic data

The population consisted of 614 women undergoing IVF/ICSI treatment followed by fresh embryo transfer on either days 2, 3, or 5. Demographic data are shown in Table 1. Overall, patients had a mean age of 32.5 ± 4.6 years and a mean BMI of 25.1± 4.2 kg/m^2^. Maternal age, basal LH, basal FSH and smoking did not differ between 17-OH P_4_ groups. Paternal age and BMI showed no significant differences between 17-OH P_4_ groups (data not shown). Maternal BMI was significantly higher in the low 17-OH P_4_ group (17-OH P_4_ <6 nmol/l) compared pairwise to any of the other 17-OH P_4_ groups (all pairwise *p* < 0.001). Antral follicle count and the distribution of women with PCOS differed significantly between groups, albeit with no apparent clinically relevant differences.

**Table 1:** Baseline characteristics of study patients in different 17-OH P_4_ groups.

**17-OH P_4_ (nmol/l)**	***N***	**All**	**<6**	**6–14**	**14.1–30**	**>30**	***p***
Number of patients, *n*	614		183	134	132	165	
Maternal age, years	614	32.5 ± 4.6	33.0 ± 4.9	32.2 ± 4.6	32.7 ± 4.5	32.0 ± 4.2	0.195
Maternal BMI, kg/m^2^	614	25.1 ± 4.2	26.3 ± 4.0	24.2 ± 4.1	24.7 ± 4.4	24.5 ± 4.1	<0.001
Maternal smoking,%	614	8	10	8	4	9	0.197
Basal FSH, IU	573^*^	6.2 (0.1–22.0)	6.1 (0.3–22.0)	6.7 (0.1–17.5)	6.4 (0.3–15.5)	6.0 (1.1–14.7)	0.544
Basal LH, IU	554^**^	5.4 (0.1–40.0)	5.2 (0.2–40.0)	5.2 (0.1–19.0)	5.4 (0.1–17.0)	5.6 (0.4–24.0)	0.792
Antral follicle count, *n*	614	13 (2–50)	12 (2–33)	14 (3–40)	12 (2–38)	13 (4–50)	0.004
Primary diagnosis, Unexplained, % Tubal, % PCO/PCOS, % Endometriosis, % Male, % Single/female partner, % Other, %	614	25 9 11 6.5 38 10 1	21 7 11 7 39 14 1	22 8 13 7 45 5 0	30 11 5 7 37 9 1	29 11 14 5 30 10 1	0.200^***^ 0.426 0.028 0.781 0.074 0.095 0.895

*Baseline characteristics are presented as mean ± SD for continuous parametric data and as median (range) for continuous non-parametric data. Categorical data is presented as percentages (%)*.

* *Data on basal FSH levels were missing in 41 patients (6.7%). Patients with missing data on basal FSH levels were equally distributed across 17-OH P_4_ groups (p = 0.49)*.

** *Data on basal LH levels were missing in 60 patients (9.8%). Patients with missing data on basal LH levels were equally distributed across 17-OH P_4_ groups (p = 0.43)*.

*** p-value describes the comparison between the chosen primary diagnosis category and the combined group of all other primary diagnosis categories. SI conversion factor for 17-OH P_4_: nmol/l = 3.03 ^*^ ng/ml.

### Cycle characteristics

A total of 63% of patients were treated in a GnRH antagonist protocol, whereas a long GnRH agonist protocol was used in 37% of patients. Final oocyte maturation was achieved using hCG trigger in 58% of patients and using GnRH agonist trigger in 42% of patients. In total, 64% of patients had a top-quality embryo for transfer (Table 2).

**Table 2:** Descriptive data of controlled ovarian stimulation, oocytes, embryo transfer, and luteal phase support.

**17-OH P_4_, nmol/l**	**All**	**<6**	**6-14**	**14.1-30**	**>30**	***p***
Number of patients (*n*)	614	183	134	132	165	
Protocol Antagonist (%) Long GnRH agonist (%)	63 37	51 49	58 42	61 39	81 19	<0.001
Total FSH dose (IU)	2,250 (500–7,350)	2,450 (900–6,750)	2,063 (788–7350)	2,475 (900–5025)	1,950 (500–7,350)	<0.001
Stim duration (days)	10.4 ± 2.0	10.4 ± 1.9	10.6 ± 2.1	10.6 ± 2.1	9.9 ± 1.9	0.006
Final follicle count >12 mm on trigger day	10 (1–29)	10 (1–29)	9 (1–22)	9 (3–20)	10 (1–26)	0.005
Mode of triggering for final oocyte maturation						
hCG (%)	58	67	72	61	35	<0.001
GnRH agonist (%)	42	33	28	39	65	
Number of oocytes retrieved (n)	8 (1–28)	9 (1–23)	8 (1–26)	8 (1–24)	9 (2–28)	0.014
Number of fertilized oocytes (n)	8 (1–27)	8 (1–23)	8 (1–23)	7 (1–24)	8 (2–27)	0.033
Single embryo transfer (%) Double embryo transfer (%)	81 19	79 21	79 21	83 17	83 17	0.661
At least one top quality embryo for transfer (%)	64	66	59	70	64	0.160
Mean embryo score, SET DET	1.4 ± 0.6 1.7 ± 0.6	1.4 ± 0.6 1.7 ± 0.6	1.5 ± 0.7 1.7 ± 0.7	1.3 ± 0.5 1.6 ± 0.7	1.5 ± 0.7 1.6 ± 0.6	0.090 0.915
Day of transfer, Cleavage-stage embryo transfer (%) Blastocyst transfer (%)	72 28	72 28	72 28	77 23	68 32	0.418
Luteal phase support Vaginal progesterone only (%) + 1 bolus of hCG (%) + 2 boluses of hCG (%) Vaginal P + Gonapeptyl (%)	52 17 25 6	61 28 4 7	66 19 8 7	53 9 30 8	31 9 56 4	<0.001^*^ <0.001 <0.001 0.353

*Descriptive data is presented as mean ± SD for continuous parametric data and as median (range) for continuous non-parametric data. Categorical data is presented as percentages (%)*.

* p-value describes the comparison between the chosen luteal phase support category and the combined group of all other luteal phase support categories. SI conversion factor for 17-OH P_4_: nmol/l = 3.03 ^*^ ng/ml.

The low 17-OH P_4_ group (<6 nmol/l) and the high 17-OH P_4_ group (>30 nmol/l) both had a significantly higher final follicle count (*p* = 0.01) and a significantly higher number of oocytes retrieved (*p* = 0.01) compared to the two remaining 17-OH P_4_ groups. Furthermore, total FSH dose, duration of stimulation and the luteal phase support regime differed between groups (Table 2).

Single embryo transfer was applied in 81% and double embryo transfer in 19% of patients. There was no significant difference between the number of embryos transferred across 17-OH P_4_ groups (*p* = 0.66) A cleavage-stage embryo transfer was performed on day 2 or 3 in 72% of patients, whereas 28% had a blastocyst transfer on day 5. The study blastocyst transfer rate is in line with the present blastocyst transfer rate for all public IVF clinics in Denmark ^2^. The percentages of patients with blastocyst transfer were comparable across the four 17-OH P_4_ groups (*p* = 0.42). Likewise, the mean embryo score was similar in different 17-OH P_4_ groups for both SET (*p* = 0.09) and DET transfers (*p* = 0.92).

### Mid-luteal 17-OH P_4_ levels

**Figure 2:**
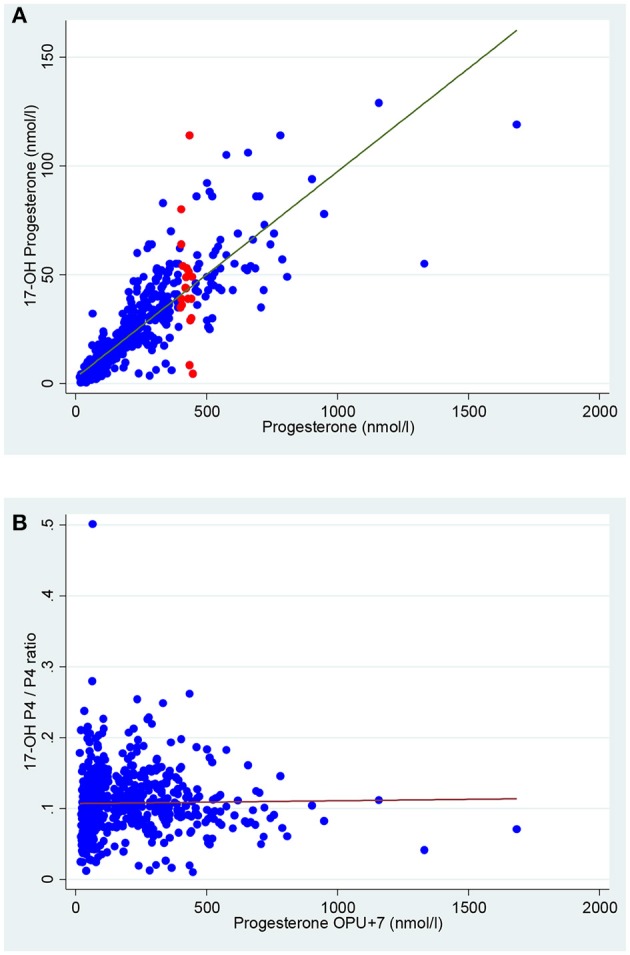
Correlation between mid-luteal serum 17-OH P4 and serum P4 in 614 women undergoing IVF treatment. **(A)** A significant positive correlation was found between serum P_4_ and serum 17-OH P_4_, *p* < 0.001.Red dots depict the 19 patients in the cohort with P_4_ 400–450 nmol/l. **(B)** The ratio between serum 17-OH P_4_ and serum P_4_ was constant at ~10% throughout the P_4_ range, *p* = 0.67. The linear regression line is marked in red.

The median 17-OH P_4_ concentration measured on OPU+7 was 13.2 nmol/l, range 0.5–129.0 nmol/l. The median P_4_ concentration was 113 nmol/l, range 16.3–1685.0 nmol/l. There was a significant, positive association between P_4_ levels and 17-OH P_4_ levels, *p* < 0.001 (Figure 2A). Thus, an increase of 100 nmol/l in serum P_4_ levels corresponded to an increase in serum 17-OH P_4_ levels of 9.5 nmol/l, 95%CI [9.0;9.9]. This ratio between serum 17-OH P_4_ and serum P_4_ of ~10% was constant with increasing levels of P_4_, *p* = 0.67 (Figure 2B). However, a large inter-individual difference in the secretion pattern of P_4_ and 17-OH P_4_ was noticed. To illustrate this, the 19 patients in the cohort with serum P_4_ values between 400–450 nmol/l are marked in red in Figure 2A. Despite comparable levels of P_4_ in these patients, the range of 17-OH P_4_ varied from levels as low as 4.4 nmol/l up to 114 nmol/l.

Patients were equally distributed in the four chosen 17-OH P_4_ groups. Out of the total cohort of 614 patients, 30% (*n* = 183) had 17-OH P_4_ levels <6 nmol/l, 22% (*n* = 134) had 17-OH P_4_ levels between 6 and 14 nmol/l, 21% (*n* = 132) had 17-OH P_4_ levels between 14.1 and 30 nmol/l and finally, 27% (*n* = 165) had 17-OH P_4_ levels >30 nmol/l. The 72 patients (12%) who were omitted from the final regression analysis due to missing values of covariates, were equally distributed across the four 17-OH P_4_ groups (*p* = 0.94).

### Reproductive outcomes

The overall rate for positive hCG per transfer was 47% (291/614), the clinical pregnancy rate per transfer was 35% (214/614) and the overall live birth rate per transfer was 34% (207/614). The early pregnancy loss rate was 26% (77/291), and the clinical pregnancy loss was 3% (7/214). The minimum and maximum levels of 17-OH P_4_ in patients with a live birth were 0.65 nmol/l and 114 nmol/l, respectively.

**Figure 3:**
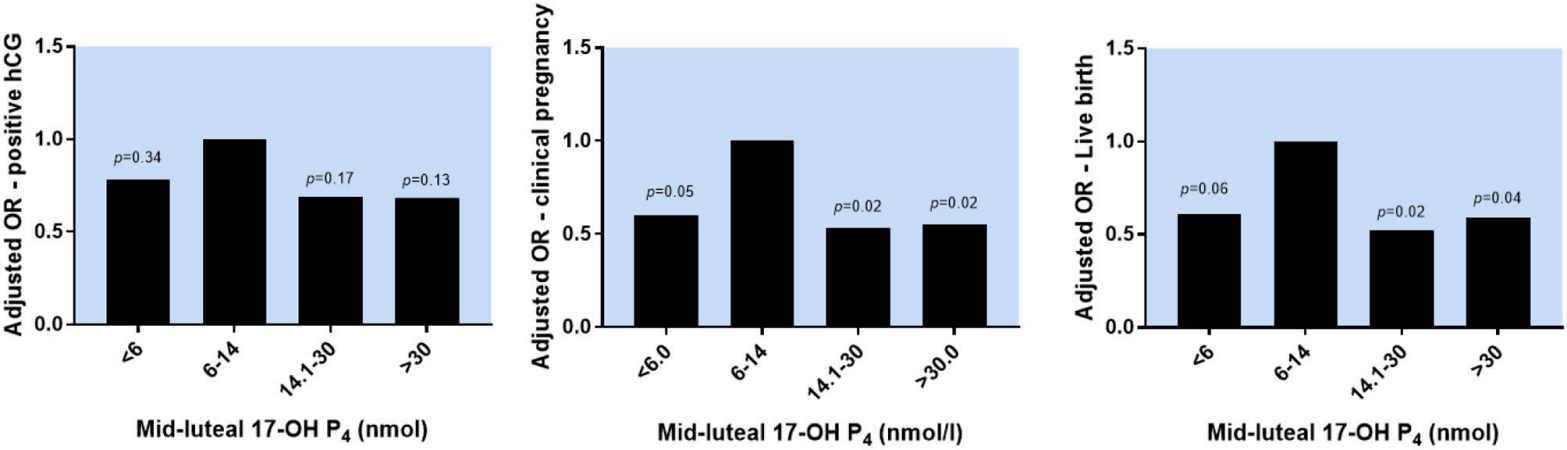
The association between mid-luteal serum 17-OH P_4_ levels and reproductive outcomes. OR for positive hCG, clinical pregnancy and live birth in different 17-OH P4 groups adjusted for maternal age, maternal BMI, day of embryo transfer, late follicular P4 levels, smoking and final number of follicles. *P*-values refer to the pairwise comparison between each 17-OH P4 category and the reference group (6–14 nmol/l).

When evaluating the association between mid-luteal 17-OH P_4_ and reproductive outcomes, the optimal serum level of 17-OH P_4_ was between 6 and 14 nmol/l. Below but also above this level, the OR for positive hCG, clinical pregnancy and live birth showed a non-linear pattern indicating a negative impact on the reproductive outcomes (Figure 3). Thus, OR for live birth in the low 17-OH P_4_ group was 0.61, 95% CI [0.36;1.01], *p* = 0.06. Likewise, above the optimal 17-OH P_4_ range the OR for live birth was significantly decreased: OR 0.59, 95%CI [0.35;0.98], *p* = 0.04. As seen from Figure 3, the association between 17-OH P_4_ and reproductive outcomes displays a non-linear pattern and the negative impact of a high mid-luteal 17-OH P_4_ level seems to be just as strong as the negative impact of low 17-OH P_4_ in the peri-implantation period.

In sensitivity analyses, adding trigger type, or protocol type to the statistical model did not change estimates significantly. Furthermore, when using 25/50/75 percentiles or 10/50/90 percentiles to define four 17-OH P_4_ groups, the same non-linear pattern for reproductive outcomes was found as seen with the a priori chosen 17-OH P_4_ groups presented above, however, with smaller differences between groups (Supplementary Figure 1).

For a reference person (30 years old, BMI 25 kg/m^2^, 8 follicles on the day of trigger, late follicular phase P_4_ ≤ 4.77 nmol/l, non-smoker) the chance of a live birth following blastocyst transfer was 53%, 95% CI [42;64%] if mid-luteal 17-OH P_4_ was within the optimal range (6–14 nmol/l). With mid-luteal 17-OH P_4_ levels above the optimal range, the chance of a live birth decreased significantly to 41%, 95% CI [31;52%], thus an absolute risk difference of −12 percentage points, 95% CI [−22%;−0.01%], *p* = 0.04. With mid-luteal 17-OH P_4_ levels below the optimal level, the chance of a live birth was 43%, 95%CI [33;53%], thus an absolute risk difference of −10 percentage points, 95% CI [−21;0.1%], *p* = 0.07.

No significant correlation between mid-luteal 17-OH P_4_ levels and early pregnancy loss was found (Table 3).

**Table 3:** Reproductive outcome in different luteal 17-OH P_4_ groups.

	**Cohort for mid-luteal 17-OH P**_**4**_ **monitoring**															
	**OR for positive hCG**				**OR for clinical pregnancy**				**OR for live birth**				**OR for early pregnancy loss**			
	***N***	**Crude OR [95% CI]**	***N***	**Final adjusted OR [95% CI]**	***N***	**Crude OR [95% CI]**	***N***	**Final adjusted OR [95% CI]**	***N***	**Crude OR [95% CI]**	***N***	**Final adjusted OR [95% CI]**	***N***	**Crude OR [95% CI]**	***N***	**Final adjusted OR [95% CI]**
	291/614	614^*^		542^**^	214/614	614^*^		542^*^	207/614	614^*^		542^**^	77/291	614^*^		535^***^
17-OH P_4_ < 6 nmol/l	88/183	0.82 [0.53;1.29]	161	0.78 [0.47;1.30]	65/183	0.74 [0.47;1.18]	161	0.60 [0.36;1.01]	62/183	0.74 [0.46;1.17]	161	0.61 [0.36;1.01]	23/88	1.23 [0.61;2.50]	160	1.82 [0.81;4.02]
17-OH P_4_ 6–14 nmol/l	71/134	1.00	120	1.00	57/134	1.00	120	1.00	55/134	1.00	120	1.00	14/71	1.00	119	1.00
17-OH P_4_ 14.1–30 nmol/l	58/132	0.70 [0.43;1.13]	115	0.69 [0.40;1.17]	41/132	0.61 [0.37;1.61]	115	0.53 [0.30;0.92]	39/132	0.60 [0.36;1.01]	115	0.52 [0.30;0.91]	17/58	1.18 [0.55;2.54]	111	1.60 [0.69;3.72]
17-OH P_4_ >30 nmol/l	74/165	0.72 [0.46;1.14]	146	0.68 [0.41;1.13]	51/165	0.60 [0.37;0.98]	146	0.55 [0.33;0.92]	51/165	0.64 [0.40;1.04]	146	0.59 [0.35;0.98]	23/74	1.32 [0.65;2.69]	145	1.49 [0.67;3.32]

* *In the crude OR estimates, all 614 patient with embryo transfer were included*.

** *Due to missing data on the covariate late follicular P_4_ level in 72 patients, the final adjusted regression model included 542 patients. Patients with missing data were equally distributed across 17-OH P_4_ groups (p = 0.94)*.

*** Due to missing data on the covariate peak follicular E_2_ level in 79 patients, the final adjusted regression model for early pregnancy loss included 535 patients. Patients with missing data were equally distributed across 17-OH P_4_ groups (p = 0.67). SI conversion factor for 17-OH P_4_: nmol/l = 3.03 ^*^ ng/ml. CI, confidence interval.

### Daytime variations in serum 17-OH P_4_ levels

**Figure 4:**
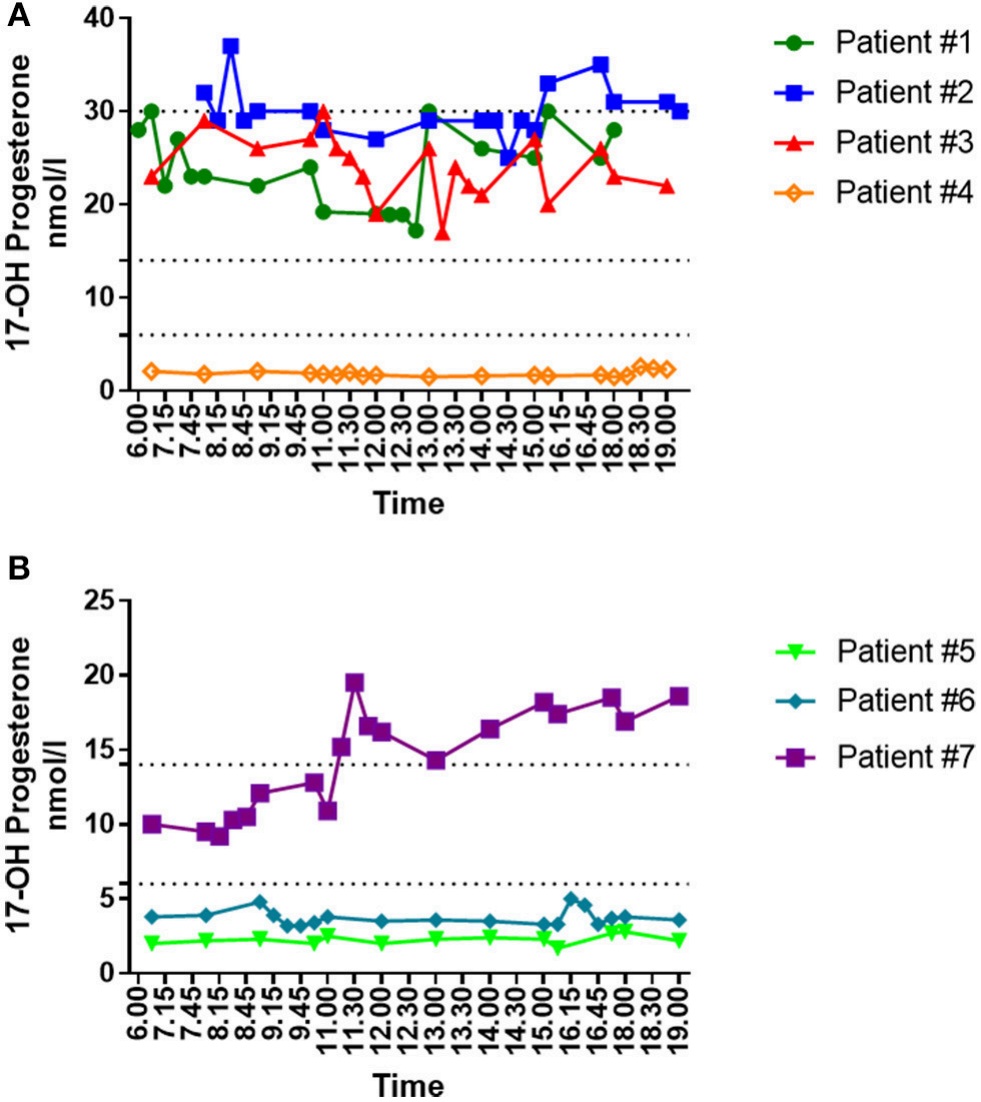
Individual daytime variation in mid-luteal serum 17-OH P_4_ levels in women undergoing IVF treatment and fresh embryo transfer. **(A)** Daytime variations in mid-luteal serum 17-OH P_4_ in four women treated in the long GnRH agonist protocol and trigged for final oocyte maturation with hCG. All patients received vaginal P_4_ for luteal phase support. Dotted lines depict 6, 14, and 30 nmol/l, respectively. **(B)** Daytime variations in mid-luteal serum 17-OH P_4_ in three women treated in the GnRH antagonist protocol and trigged for final oocyte maturation with a bolus of GnRH agonist. All patents received vaginal P_4_ for luteal phase support in combination with one (patient#5 and #6) or two (patient #7) boluses of hCG on the day of oocyte pick-up and 5 days later, respectively. Dotted lines depict 6 and 14 nmol/l.

Figure 4 shows the individual daytime variations in mid-luteal serum 17-OH P_4_ concentration in seven women undergoing IVF treatment. Three of these women (#4, #5, and #6) had very low endogenous 17-OH P_4_ production with median concentrations during daytime between 1.9 and 3.8 nmol/l compared to 13.2 nmol/l for the total study cohort. It is seen from Figure 4 that in patients with diminished luteal phase 17-OH P_4_ production (< 6 nmol/l), serum concentrations of 17-OH P_4_ displayed a constant pattern though out daytime without any significant fluctuations. In contrast, in patients with 17-OH P_4_ levels above 6 nmol/l, sudden fluctuations in 17-OH P_4_ occurred randomly in different patients without any obvious common pattern. In patient #1, 17-OH P_4_ increased 12.8 nmol/l in just 15 min (12.45–13.00 p.m.). This rise in concentration corresponds to an increase of 53% compared to the median level for the day, and this rise occurred even though LH levels were below the detection limit throughout the study period (LH data not shown).

## Discussion

This prospective study, including 614 women undergoing IVF and fresh embryo transfer, aimed at investigating whether the mid-luteal serum 17-OH P_4_ concentration–used as an index of corpus luteum (CL) function–affects the reproductive outcome. The results suggest that positive hCG rates, clinical pregnancy rates, and live birth rates are reduced outside the defined optimal range for 17-OH P_4_ (6–14 nmol/l). Furthermore, for the first time in IVF patients, we monitored the variation in mid-luteal serum 17-OH P_4_ levels showing that patients with diminished CL function displayed a constant hormone pattern without any significant daytime fluctuations in serum 17-OH P_4_ concentrations.

The CL produces 17-OH P_4_, as well as, P_4_ during the luteal phase (13). However, the secretion pattern of the two steroids differs. Coinciding with the LH peak, an initial distinct 17-OH P_4_ peak occurs reflecting the initial luteinisation and growth of the theca lutein cells—the luteal cell-line capable of 17-OH P_4_ synthesis (13). After 2–4 days of decline, the 17-OH P_4_ levels increase again—now in parallel with P_4_ reaching a second peak during the mid-luteal phase, followed by a decrease toward the end of the luteal phase. In the natural cycle, the mid-luteal ratio of 17-OH P_4_/P_4_ is reported to be 10–20% (13, 23–26). We found a similar ratio of ~10% in our cohort and this ratio did not change significantly with increasing levels of P_4_. The large inter-individual differences seen in the secretion pattern of P_4_ and 17-OH P_4_ underline that the CL function is highly individual, and that comparable values of P_4_ in individual patients may correspond to very diverse levels of 17-OH P_4_. Furthermore, three out of seven randomly chosen patients who participated in the daytime monitoring displayed severely reduced endogenous 17-OH P_4_ levels throughout the day (median levels 1.9–3.8 nmol/l). These concentrations are even lower than seen during the mid-luteal phase of the natural cycle (~6 nmol/l) (18). Additionally, the very low serum 17-OH P_4_ levels were accompanied by low serum P_4_ levels (36–55 nmol/l). The exogenous vaginal P_4_ supplementation induces a serum P_4_ level of ~30–40 nmol/l, thus underlining that the abovementioned three patients had a severely diminished endogenous P_4_ secretion. This occurred although two of the patients had 17 and 19 follicles, respectively, on the day of trigger. Thus, the CL function is individual, and a large number of CLs do not necessarily warrant a high steroid output in the mid-luteal phase. Furthermore, it seems that a severely decreased mid-luteal CL function is not a rare finding following IVF treatment despite a sufficient trigger regimen and luteal phase support.

The biological effect of 17-OH P_4_ is not well-described. Whereas, P_4_ has a fundamental impact on the decidualization process (27), the maternal immunological adaption in early pregnancy (28) and the dampening of uterine contractions at the time of implantation (29), the endogenous 17-OH P_4_ has only very weak progestogen effects (30). The binding affinity of 17-OH P_4_ to both P_4_ receptors (PR-A and PR-B) is only 1% of that of P_4_. Furthermore, upon binding, the capacity of 17-OH P_4_ to activate subsequent gene expression is very low and only ~0.12% of that of P_4_ (30). Thus, even though P_4_ and 17-OH P_4_ are structurally similar and are secreted in parallel from the CL, they seem to work in different ways. In serum, P_4_ is tightly bound to cortisol-binding protein (18%) and loosely bound to albumin (80%) whereas only 2% of P_4_ is unbound (free) (24, 31). The free form of P_4_ is available for diffusion out of capillaries, into cells where it exerts its function (32). A fraction of the secreted P_4_ and 17-OH P_4_ from the CL is transported directly to the uterus through a counter-current exchange mechanism from the utero-ovarian veins into the utero-ovarian arteries driven by a large concentration gradient (33, 34). This mechanism may function to secure a high biological steroid concentration from the site of production (the ovaries) directly to the target organ (the endometrium) (34, 35). Another fraction of secreted P_4_ and 17-OH P_4_ from the CL enters circulation directly via the ovarian veins, which terminate in the inferior vena cava (right) and the renal vein on the left (36). The binding affinity of 17-OH P_4_ to cortisol-binding protein (CBP) is much greater than that of P_4_ and close to that of cortisol (24). It can be speculated that 17-OH P_4_ acts by displacing P_4_ and cortisol from CBP, thereby increasing the free active hormone concentration locally in the ovarian veins. Thus, this mechanism will ensure a high, free P_4_ concentration facilitating the counter-current transport from the venous to the arterial vascular bed and hence, an increased direct transport of P_4_ to the endometrium.

It should be emphasized that natural, endogenous 17-OH P_4_ differs chemically and biologically from the synthetic progestin 17-OH P_4_ caproate (17-OHPC). The latter is a synthetic progestogen (compound with progesterone-like action) and is not produced endogenously (37). The 17-OHPC binds more avidly to the P_4_ receptor than natural 17-OH P_4_, eliciting a sustained and robust progestogen effect on the endometrium (30). Thus, 17-OHPC can be used as luteal phase support (IM administration) whereas monotherapy with natural 17-OH P_4_–with a very weak direct progestogen effect—probably would be inefficient in terms of rescuing the luteal phase following IVF treatment.

From a clinical viewpoint, 17-OH P_4_ may be used as a direct biomarker for luteal phase function, as 17-OH P_4_ is not supplied as part of the luteal P_4_ supplementation regimen. Thus, the measured 17-OH P_4_ reflects the endogenous production predominantly from the CL, as only a minor fraction (~0.5 nmol/l) of circulating mid-luteal 17-OH P_4_ originates from the adrenal glands (23, 26).

Our findings of a non-linear association between 17-OH P_4_ levels and the reproductive outcomes is in line with other studies examining luteal phase steroid profiles. Following frozen-thawed embryo transfer, work by Yovich et al. (8) and Alsbjerg et al. (10) both showed a diminished chance of ongoing pregnancy if serum P_4_ was above or below a defined optimal P_4_ range. Similarly, in a previous paper using the present patient cohort, we found a consistently non-linear pattern describing the association between early and mid-luteal P4 levels and reproductive outcomes. Thus, suggesting that both low, as well as, high luteal P4 levels reduce the chance of a positive pregnancy outcome following fresh embryo transfer (9). In that study, P_4_ monitoring was performed during the early luteal phase (2–3 days following OPU) or in the mid-luteal phase (OPU+5). The same pattern emerged in this study, measuring serum 17-OH P_4_ on OPU+7.

Taken together, the non-linear pattern between luteal steroid levels and reproductive outcomes seems to apply both to P_4_ and 17-OH P_4_, to different days in the luteal phase (2, 3, 5, or 7 days after OPU) and to both the fresh and frozen embryo transfer cycle (8–11). Furthermore, the above-mentioned studies all found a consistency in the absolute risk reduction (14–20 percentage points) below or above the defined P_4_ which is in line with our present results.

In this study, daytime monitoring of 17-OH P_4_ showed that patients with a diminished luteal phase function (17-OH P_4_ <6 nmol/l) displayed a constant 17-OH P_4_ pattern throughout daytime without any significant fluctuations in serum levels. Thus, measurement of luteal 17-OH P_4_ concentrations will accurately detect patients with low endogenous 17-OH P_4_ levels and, thus, a decreased corpus luteum function. In patients with higher 17-OH P_4_ concentrations, fluctuations in serum 17-OH P_4_ concentrations occur in a random fashion without any obvious common pattern between patients.

The possible clinical effect of serum fluctuations is demonstrated in patient #7 (Figure 4). When measuring 17-OH P_4_ levels at 8.00 a.m., the patient would be classified in the optimal 17-OH P_4_ range between 6 and 14 nmol/l. However, if measurements were performed at 12.00 p.m., she would be categorized in the 14.1–30 nmol/l group. Similarly, patient #2 shift between 17-OH P_4_ group 14.1–30 and >30 nmol/l depending on the time of measurements. Thus, the figure demonstrates that when 17-OH P_4_ monitoring is done 7 days after OPU, there is a risk of misclassification of patients if serum 17-OH P_4_ >6 nmol/l. The finding, that the magnitude of the 17-OH P_4_ fluctuations depends on the 17-OH P_4_ concentration, is in total agreement with the P_4_ daytime variation on OPU+7 previously shown by our group (14).

We monitored 17-OH P_4_ on OPU+7 to explore whether the non-linear association between progestogen levels and reproductive outcome was still present at the time of implantation compared to earlier luteal measurements (2, 3, or 5 days following OPU) (9). Based on the present results, this seems to be the case. Performing the luteal monitoring early in the luteal phase, allows for an intervention based on the results. Thus, better reproductive outcomes may be obtained by additional exogenous luteal P_4_ support to the low P_4_ or 17-OH P_4_ group and by segmentation followed by subsequent embryo transfer in a frozen/thawed cycle in case of a high P_4_ or 17-OH P_4_ level. In a clinical setting monitoring of P_4_ or 17-OH P_4_ on OPU+7 is disadvantageous as the clinical consequence of a “too low” or “too high” progestogen level is limited. Thus, at this time of cycle, the embryo is already transferred and the effect of administering additional exogenous P_4_ during peri-implantation may be reduced compared to administration earlier in the luteal phase (38). Furthermore, in some IVF patients, peak levels of P4 and 17-OH P4 are seen already on day 5–6 and following this P4 and 17-OH P4 start to decrease. It could be hypothesized that some of the patients with low 17-OH P4 measured on day 7, had sufficient levels of 17-OH P4 earlier in the luteal phase and therefore are classified as “false low” on day 7. These patients could theoretically belong to a group with better pregnancy chance compared to patients with consistently low 17-OH P4 levels throughout the luteal phase. This misclassification of some of the patients could potentially affect the OR in the low 17-OH P4 group and underestimate the effect of low 17-OH P4 on the chance of pregnancy.

Whether the 17-OH P_4_ monitoring offers a clinical advantage compared to the more traditional P_4_ monitoring may be questioned. The 17-OH P_4_ does not seem to display a more stable luteal daytime pattern in patients with sufficient CL function compared to P_4_ (14). Furthermore, whereas the analytical performance of P_4_ immunoassays is generally high, the immunoassays available for the quantification of 17-OH P_4_ suffer from important analytical limitations (39, 40). The specificity of 17-OH P_4_ measured by immunoassays is critically limited due to reduced reproducibility and cross reactivity with particularly P_4_ (40). To account for this, 17-OH P_4_ quantification must be performed using LC-MS/MS to obtain sufficient accuracy. This requires more manual work for the medical laboratory technician compared to a P_4_ quantification using standard immunoassays and this more than triples the expense per sample. Finally, as demonstrated by Figure 2A, some patients display a low mid-luteal 17-OH P_4_ level even though the concomitant measured P_4_ level seems sufficient. This phenomenon may reflect an isolated defect in the function of the luteinized theca cells but a sufficient P_4_ output from the luteinized granulosa cells (13) and may lead to a misclassification of the patient.

The key strengths of the present study include its prospective design, the large cohort of patients and the systematic approach to the handling of confounding factors by use of Directed Acyclic graphs minimizing the risk of collider stratification (41). Furthermore, all patients received the same type and dose of vaginal P_4_ supplementation in the luteal phase ensuring a basis for comparison between patients. Furthermore, the participants included in the study were unselected broadening the generalizability of the findings.

In conclusion, this study shows for the first time that the chance of a live birth is reduced by ~10 percentage points below, but also above the defined optimal range for 17-OH P_4_ measured on OPU+7. This finding supports the emerging evidence that the absolute concentrations of luteal P_4_ seem to affect the reproductive outcomes following IVF treatment. Based on the present study, luteal monitoring of 17-OH P_4_ levels alone does not seem to offer a better insight into the CL function compared to the monitoring of total P_4_ levels.

## Author contributions

LT, PH, CA, and KE designed the study. LT drafted the manuscript and UK, CA, KE, MO and PH all contributed to the interpretation of data and critically reviewed the manuscript. All co-authors participated in the conduction of the study and approved the final manuscript.

### Conflict of interest statement

LT received an unrestricted research grant from Ferring Pharmaceuticals to support this work. PH received unrestricted research grants from MSD, Merck, and Ferring Pharmaceuticals, as well as, honoraria for lectures from MSD, Merck, and Gedeon Richter outside of this work. USK received honoraria for lectures from MSD and Ferring Pharmaceuticals outside of this work. CA received unrestricted research grants from MSD, IBSA, and Ferring Pharmaceuticals, as well as, honoraria for lectures from MSD and IBSA outside of this work. The remaining authors declare that the research was conducted in the absence of any commercial or financial relationships that could be construed as a potential conflict of interest.

## Footnotes

^1^Danish Fertility Society. *Guideline 2016*: Overweight, Obesity and Fertility Treatment.

^2^Danish Fertility Society. Annual Report 2017.
