# Supplementary material for: Mid-Luteal 17-OH Progesterone Levels in 614 Women Undergoing IVF-Treatment and Fresh Embryo Transfer—Daytime Variation and Impact on Live Birth Rates
Source: Front Endocrinol (Lausanne). 2018 Nov 29;9:690. doi: 10.3389/fendo.2018.00690 (PMC6282693; doi:10.3389/fendo.2018.00690)
Supplement: Supplementary file 1 [file Data_Sheet_1.docx]

**Supplementary figure 1.**

**Supplementary Figure 1**. Graph bar plot on raw data used to define 17-OH P_4_ groups for the final regression analysis (A). Adjusted OR for clinical pregnancy rate in study 17-OH P_4_ groups (B). Adjusted OR for clinical pregnancy by use of 25/50/75 percentiles (C) and by use of 10/50/90 percentiles (D). *p*-values refer to the pairwise comparison between each 17-OH P_4_ category and the reference category. * *p*<0.05

**Supplementary figure 2.**


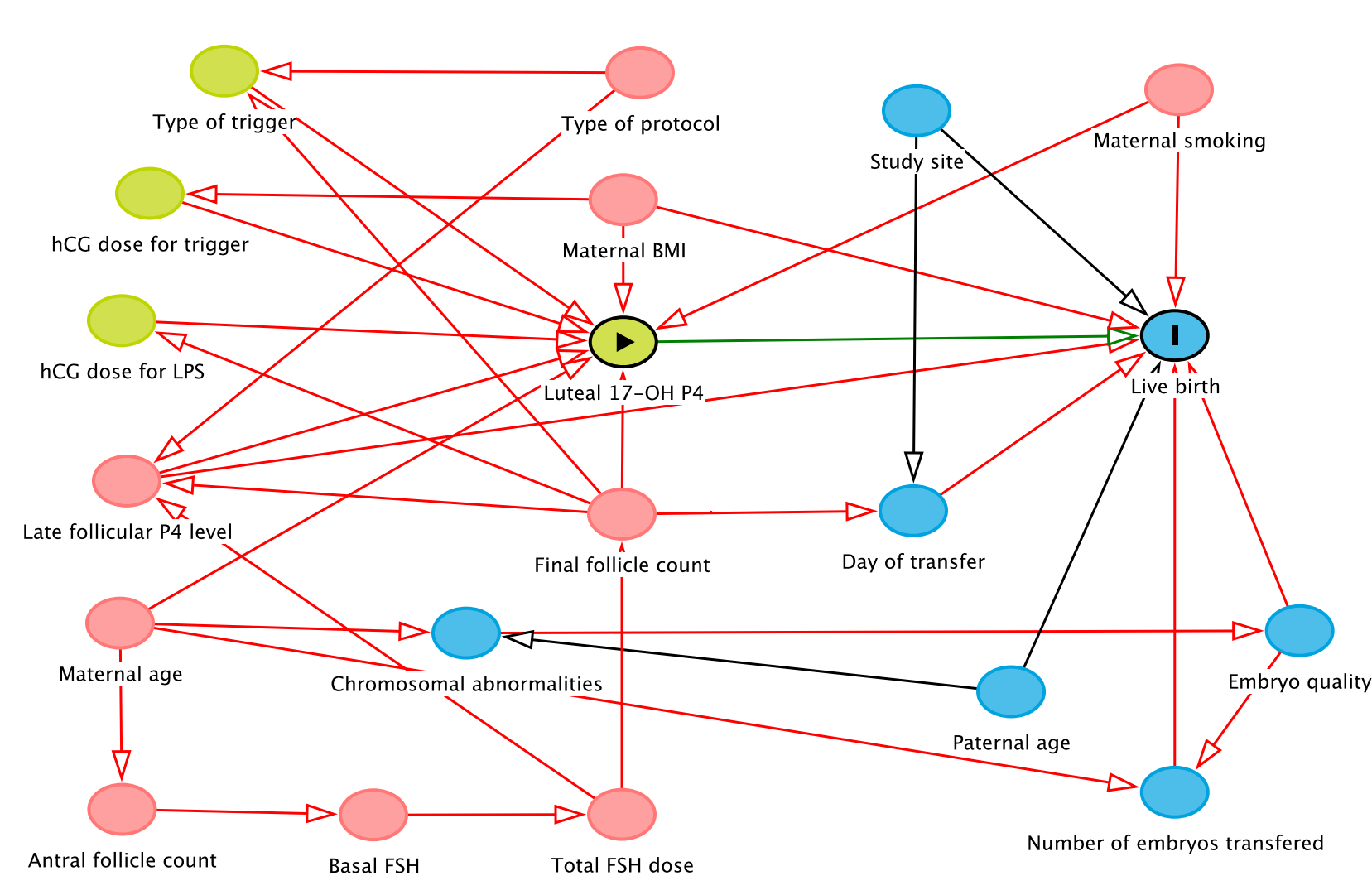


**Supplementary Figure 2.** Directed Acyclic Graph (DAG) showing the association between mid-luteal 17-OH P_4_ and live birth and the possible confounding pathways with relevance for the association. The causal path between exposure (luteal 17-OH P_4_) and outcome (live birth) is depicted in green. Red circles represent ancestors of both exposure and outcome. Blue circles represent ancestors of the outcome. Green circles represent ancestors of the exposure. Red connections show biasing pathways.
